# Should Marijuana Be Legalized: A Scoping Review of Associations of Marijuana and Depression

**DOI:** 10.7759/cureus.42835

**Published:** 2023-08-02

**Authors:** Prathma Anandbhai Dave, Ralph Kingsford Rohit, Charu Tibrewal, Naisargi Shrikant Modi, Parth S Bajoria, Siddharth Kamal Gandhi, Priyansh Patel

**Affiliations:** 1 Department of Internal Medicine, Medical College Baroda, Vadodara, IND; 2 Department of Internal Medicine, Dayanand Medical College and Hospital, Ludhiana, IND; 3 Department of Internal Medicine, Civil Hospital Ahmedabad, Ahmedabad, IND; 4 Department of Internal Medicine, Gujarat Medical Education and Research Society Gandhinagar, Gandhinagar, IND; 5 Department of Internal Medicine, Shri M. P. Shah Medical College, Jamnagar, IND

**Keywords:** ethic, therapeutic use, marijuana use, depression, substance abuse

## Abstract

People with addiction to marijuana and those who have ever consumed marijuana at any time during their life suffer from depression at some point in their life. Depression has been associated with substance use as both a trigger and repercussion. A total of 3663 articles were analyzed, and 26 articles were collectively selected for this study. Consuming marijuana was linked to the development of depression in the majority of individuals. Marijuana consumption and its repercussions have both been connected to negative effects on the body, such as respiratory disorders and even psychological disorders, including stress and depressive disorders. Studies potentially point to a complicated causal relationship between marijuana consumption and depressive disorder, stating that early depressive symptoms enable marijuana usage, which then reduces depression. A research article clearly states that consuming marijuana can be helpful in elevating mood and anxiolytic initially, but it is subsequently followed by a rise in depressive symptoms, which manifest as mental distress and frustration. Discussions with patients about the extent of their marijuana consumption, techniques for reducing the use, and the impact of marijuana on depression may be beneficial in medical facilities where depressive disorder is treated. This research paper highlights the importance of understanding depression and the use of marijuana for temporary relief from depressive symptoms and its long-term consequences on mental health.

## Introduction and background

As reported by the United Nations Office on Drugs and Crime (UNODC, 2012) and World Health Organization (WHO, 2014), marijuana is the most broadly used illicit drug in the world, and in the United States (US), 30% of users have developed an addiction to marijuana [1]. In the US, marijuana ranks third in terms of drug use (after alcohol and nicotine) [2]. According to national epidemiological statistics, 47% of people with addiction to marijuana and 29% of people who have ever consumed marijuana anytime during their life met the criteria for major depressive disorder (MDD) [3]. Marijuana consumption is associated with abuse in 50-70% of those who have depression and alcohol use disorder, and affected individuals display a greater degree of depressive symptoms and psychological, cognitive, and physical deterioration [4]. According to the National Institute of Mental Health (NIMH, 2014a), mood disorders, such as depression and anxiety, are among the most prevalent mental health illnesses in the US, so knowing the contributing factors that impact them is explicitly important from a clinical outlook. Although the definite workings of these effects are yet unidentified, they appear to be restricted to cannabidiol and not delta-9-tetrahydrocannabinol (THC) [2]. People frequently reported lowered levels of emotional distress, such as depression or stress, as a reason for marijuana consumption, but a number of people also frequently report increased levels of these symptoms as a side effect of using marijuana [1]. The degree of substance abuse and depression exhibit distinguished variations by age and reach their highest point during youth [3].

Depression has been correlated with substance use as both a trigger and a consequence [5]. Developing cultural perceptions of marijuana has resulted in the legalization of its usage for therapeutic and leisurely applications in a few states of the US [6]. Treatment of symptoms associated with depression is one of the most frequent health benefits mentioned in web advertisements for commercial stores of marijuana. Moreover, advertising regarding marijuana has changed in recent years to become more favorable and contain fewer details concerning hazards. It appears that these messages are becoming increasingly common. Hence, understanding historical trends in the relationship between marijuana usage and depression has become of utmost importance given the changes in marijuana consumption and the media's portrayal of marijuana as helpful for a variety of health issues, including depressive disorder [7]. However, the predictability of the role of marijuana in the development of depression and the worsening of the symptoms with its chronic use is yet to be determined. In addition, there is limited understanding in previous research regarding the facts about how marijuana consumption can escalate the possibility of depressive disorders. In this literature review, we aim to explore if there is a bidirectional association between marijuana usage and depression and what effects it will have on the development of depressive disorder.

Methodology

Collaborating with all the authors of this paper, archives from PubMed Central, MEDLINE, and PubMed Central were assessed. The keywords from the research topic, i.e., "depression AND marijuana," were searched, and we found 3663 articles. Following this, we explored the Medical Subject Headings (MeSH) glossary and then created a search strategy ("Marijuana Use"[Majr]) AND ("Depression"[Majr]), and we were finally left with a total of 79 articles. There were no time constraints. Any identical articles and studies unconnected to the human species were promptly eliminated. Moreover, publications that were not in the English language were excluded. The results were then screened for text availability, titles, and abstracts, and the articles with full free-text reviews were selected. This narrowed down the results to 42 articles. Thereafter, the articles were analyzed, and a total of 26 articles were collectively selected for the study. Out of these, two were randomized control trials, six were research articles, 10 were observational studies, four were review articles, and four were meta-analyses.

## Review

Introduction to depression

The manifestations of depression include emotions of melancholy, dejection, and loss of enjoyment in routine tasks [8]. Poor mental state, lack of reasoning, diminished voluntary function, cognitive dysfunction, and bodily symptoms, such as lack of exuberance, sleeplessness or somnolence, a decrease or increase in weight, cognitive provocation, tiredness, and self-harm, are frequently observed medical symptoms of depressive disorders. It is widely recognized that the existence of self-harm behaviors among the sufferers of depression is linked to the seriousness of the disease, and patients with the serious disease will experience severe discomfort. They believe that their existence has no purpose, grow emotions of negativity and stress, and tend to alleviate their agony through thoughts of self-destruction [9]. In nations with advanced economies, such as the US, the financial implications resulting from depressive disorders presently surpass 80 billion dollars each year [10].

Demographics

According to internationally recognized statistics that evaluate the range of impairments resulting from each and every preeminent illness, including cardiovascular and cerebrovascular accidents, MDD stands fourth in terms of the global disability effect. A proportion of adolescents (18%) reported depressive disorders [11]. A lower percentage of men are documented to have experienced the signs of depression than women. One of the reasons for the lower percentage could be due to lesser reporting of depression in men due to cultural and societal norms. The difference in the number of cases of depression between men and women was 15%. Every 25 in 100 women are diagnosed with this disorder, and one-tenth of the male population presented with the symptoms. The number of cases and severity of depression worsened with advancing years in men and women [11].

According to one study, approximately six out of 100 men and around 10 out of 100 women are likely to suffer a bout of depression at some point in their lifespans [12]. Lately, as an increasingly prevalent mental health condition, depression has been steadily receiving recognition in the society, particularly amid the newly discovered SARS-CoV-2 respiratory infection outbreak, during which incidence of this condition rose drastically, with the WHO stating a 25% rise in the total number of cases of mood disorders during the pandemic [9]. Depressive disorders are prevalent among senior citizens; a meta-analysis demonstrated that the average frequency of symptoms associated with clinical depression is a little over 17% among people 75 years and above and almost one-fifth among people 50 years of age and above [13]. Perinatal depression is one of the widespread problems with reproduction, characterized by serious bouts of depression throughout the gestation period and/or within the first year after delivery. The frequency of perinatal depressive disorder affects roughly 10-15% of women in developed nations, with a higher frequency in developing nations. According to one study, over 20% of pregnant women suffer from depression [14].

Risk factors

**Figure 1:**
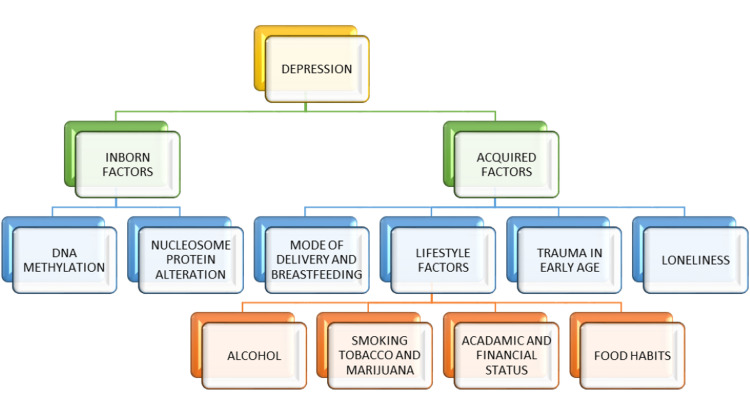
Risk factors for depression Image credits: Prathma Anandbhai Dave, Priyansh Patel, and Ralph Kingsford Rohit

There are various causes contributing to the development of depression: inborn and acquired. Alterations and diversity in the genome have been proven to be considerably unneglectable, influencing determinants in the initiation and advancement of depressive symptoms among inherited causes. From a phenotypic standpoint, DNA methyl group addition and nucleosome protein alteration play a crucial part. The mode of delivery of infants and their nutritional methods, eating behaviors, impressions from childhood, financial and academic accomplishments, and loneliness during the outbreak of the pandemic all played a role in the development of depressive symptoms [9]. Smoking tobacco products has also been correlated with a higher chance of developing depressive disorders [15]. The risk of development of depression with daily alcohol use was seen among youths. Consuming marijuana was linked to the development of depression in the majority of individuals with their ages ranging from 12 to 31 [3]. Early life events have a significant impact on depressive disorders, and adverse events in youth are regarded to be an important contributory cause for the development of depressive disorders in adults. Research has indicated that modifiable factors, such as eating junk foods and low literacy levels, have led to the development of depressive symptoms [9]. Moreover, the inability to sleep is shown to have a connection with an elevated risk of developing mood disorders [12]. Depressive symptoms when experienced for the first time in older age seem to have a less favorable outcome than in early years. First, extremely critical causes, which include grief, loneliness, disability, and physical illnesses, are quite common among elderly individuals [13]. Figure 1 shows the pictorial representation of risk factors for depression.

Diagnosis

**Figure 2:**
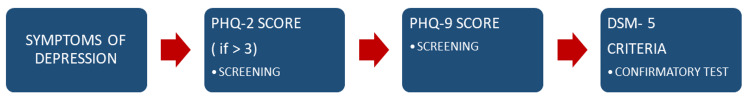
Flowchart for the diagnosis of depression Image credits: Prathma Anandbhai Dave, Priyansh Patel, and Ralph Kingsford Rohit

Every screening has to be done with suitable protocols set up to guarantee precise evaluation, successful therapy, and timely subsequent visits. Patient Health Questionnaires (PHQs) are routinely used as standardized assessment measures, which include PHQ-2 and PHQ-9. PHQ-2 and PHQ-9 comprise two and nine questions, respectively. Both can be used for assessment, but PHQ-2 has fewer false negatives and PHQ-9 has fewer number of false positives. If the result of PHQs is positive for a depressive disorder, then a confirmatory test is required to verify the result from the criteria of the Diagnostic and Statistical Manual of Mental Disorders, Fifth Edition (DSM-5) [16]. Figure 2 shows a flowchart for the diagnosis of depression. Table 1 shows PHQ-9 [17]. Table 2 shows the DSM-5 criteria for diagnosing MDD [18].

**Table 1:** Patient Health Questionnaire-9 This questionnaire was made based on the PHQ screeners [17].

Patient Health Questionnaire-9 (PHQ-9)				
Over the last two weeks, how often have you been bothered by any of the following problems?	Not at all	Several days	More than half the days	Nearly every day
Little interest or pleasure in doing things	0	1	2	3
Feeling down, depressed, or hopeless	0	1	2	3
Trouble falling or staying asleep or sleeping too much	0	1	2	3
Feeling tired or having little energy	0	1	2	3
Poor appetite or overeating	0	1	2	3
Feeling bad about yourself – or that you are a failure or have let yourself or your family down	0	1	2	3
Trouble concentrating on things, such as reading the newspaper or watching television	0	1	2	3
Moving or speaking so slowly that other people could have noticed? Or the opposite – being so fidgety or restless that you have been moving around a lot more than usual	0	1	2	3
Thoughts that you would be better off dead or hurting yourself in some way	0	1	2	3

**Table 2:** DSM-5 criteria for major depressive disorder These criteria were made based on guidelines for adult major depressive disorder [18]. DSM-5: Diagnostic and Statistical Manual of Mental Disorders, Fifth Edition

DSM-5 Diagnostic: Major Depressive Disorder		
Disease	Diagnostic criteria	Symptoms for diagnosis
Major depressive episode	Five (or more) of the symptoms have been present during the same two-week period and represent a change from previous functioning; with at least one of the symptoms being either (1) depressed mood or (2) loss of interest or pleasure. Note: Do not include symptoms that are clearly attributable to another medical condition.	Depressed most of the day, nearly every day as indicated by the subjective report (e.g., feels sad, empty, hopeless) or observations made by others (e.g., appears tearful)
		Markedly diminished interest or pleasure in all, or almost all, activities most of the day, nearly every day (as indicated by subjective accounts or observations)
		Significant weight loss when not dieting or weight gain (e.g., change of more than 5% of body weight in a month) or decrease or increase in appetite nearly every day
		Insomnia or hypersomnia nearly every day
		Psychomotor agitation or retardation nearly every day (observable by others, not merely subjective feelings of restlessness or being slowed down)
		Fatigue or loss of energy nearly every day
		Feelings of worthlessness or excessive or inappropriate guilt (which may be delusional) nearly every day (not merely self-reproach or guilt about being sick)
		Diminished ability to think or concentrate, or indecisiveness, nearly every day
		Recurrent thoughts of death (not just fear of dying), recurrent suicidal ideation without a specific plan, or a suicide attempt or a specific plan for committing suicide
	The symptoms cause clinically significant distress or impairment in social, occupational, or other important areas of functioning.	-
	The episode is not attributable to the physiological effects of a substance or to another medical condition.	-
	The occurrence of the major depressive episode is not better explained by schizoaffective disorder, schizophrenia, schizophreniform disorder, delusional disorder, or other specified and unspecified schizophrenia spectrum and other psychotic disorders.	-
	There has never been a manic episode or a hypomanic episode. Note: This exclusion does not apply if all of the manic-like or hypomanic-like episodes are substance induced or are attributable to the physiological effects of another medical condition.	-

Treatment 

Recent research suggests two main treatment modalities, which are pharmacotherapy and psychotherapy. Usually, medical professionals prefer to give pharmacotherapy, while 75% of patients prefer psychotherapy (cognitive behavioral therapy and interpersonal psychotherapy). The outcome of integrated therapy (pharmacotherapy and psychotherapy combined) was demonstrated to be advantageous as compared to behavioral therapy or drug treatment alone. Cognitive behavior therapy (CBT) is a type of psychotherapy that focuses on modifying behaviors by addressing abnormal thinking patterns over the course of multiple visits [19,20]. Monoamine reuptake inhibitors (selective serotonin reuptake inhibitors (SSRIs), serotonin-norepinephrine reuptake inhibitors (SNRIs), or tricyclic antidepressants (TCAs)) in adjunct with alpha-2 adrenergic receptor antagonists appear to be the most efficacious and acceptable combination pharmacotherapy in moderate to severe depression where monotherapy is not effective [21]. The only two drugs recognized by the US FDA to treat severe depression in youngsters and teenagers are fluoxetine (Prozac) and escitalopram (Lexapro). Fluoxetine is permitted for individuals aged ≥eight years, whereas escitalopram is recommended for those ≥12 years [20]. Figure 5 shows a variety of treatment options based on the severity of depression.

**Figure 3:**
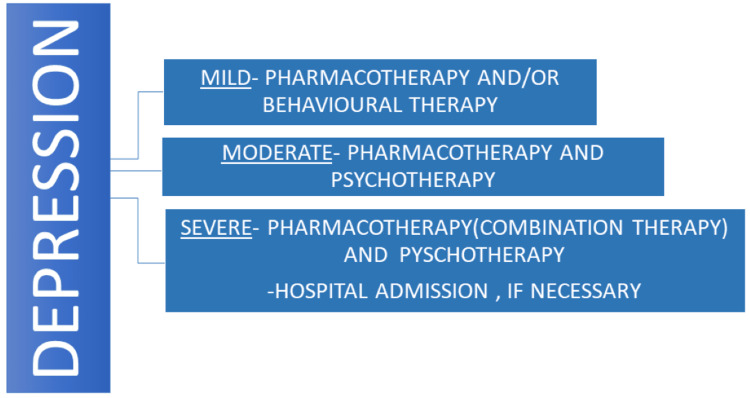
Treatment based on the severity of depression Image credits: Prathma Anandbhai Dave, Priyansh Patel, and Ralph Kingsford Rohit

Association of cannabis use and depression

Frequent marijuana usage has been linked to mediocre educational performance, such as missing classes, taking more time to graduate, obtaining below-average scores, and performing poorly in school [22]. Habitual marijuana consumption is also related to intellectual decline, including issues with decision-making capacity, sensorimotor rate, recall, understanding, comprehensive rate, and long-term focus, with certain abnormalities persisting despite cessation of its use [22]. Marijuana consumption and its repercussions have both been connected to negative effects on the body, such as respiratory disorders and psychological disorders, including stress and depressive disorders [22]. Marijuana is thought to affect the anatomy and functioning of the brain by suppressing and desensitizing neuronal endocannabinoid receptors that are abundant in the frontal lobe, white matter abnormalities, and extrapyramidal motor system. Key areas in this circuit, such as the striatum, the primary frontal cortex including the anterior cingulate cortex, and the thalamus, play a crucial role in combining data related to reward, motivation, and long-term objectives to direct appropriate and adaptive actions. These endocannabinoid receptors mainly help with synaptogenesis. Moreover, it reduces the brain adaption capacity causing both acute and chronic effects [3,23]. It is yet to be determined whether marijuana consumption raises the likelihood of depressive symptoms.

It has been proposed that the primary compounds in marijuana, THC and cannabidiol, may influence serotonin via receptors in midbrain central gray matter and other chemical messengers and cause acute effects, such as reducing stress and causing elation, but ultimately cause depressive symptoms [2,24]. However, there is not much data to back up this literal impact of marijuana use. Some additional possibilities are that marijuana consumption leads to specific adverse impacts in life, for example, reduced interest in studies, which, later on, may increase the possibility of joblessness and subsequently lead to the development of depressive disorders, or another explanation might be that the associations seen are a consequence of intersecting contributory factors, such as the family history of mental illnesses, which might raise the danger for both marijuana consumption and depressive disorder [24].

Marijuana is frequently used for its soothing effect and also its ability to elevate the mood; however, the acute psychedelic effects differ greatly [1]. Drug and alcohol abuse and instances of depressive disorder both exhibit significant chronological age patterns and spikes in youth. Because of the basic functional and interpersonal ways of development that take place during the teenage years, it appears that the correlations between drinking alcohol, drug abuse, and depressive disorder are also higher during youth [3]. Furthermore, individuals who began daily marijuana consumption during the early years of life reported more symptoms associated with depression [22]. One study suggests that long-term and excessive use of marijuana subsequently can cause depressive disorder [25]. Now, as various research indicates differences in the short-term and long-term effects of marijuana and as marijuana consumption is increasing trends, nowadays, it has become of utmost importance to have more accurate data on this topic.

Short-Term Effects of Marijuana

Harm avoidance (HA) is defined by increased nervousness, insecurity, negativity, and behavioral inhibition. Most researchers suggest that it is positively linked with depressive disorders and levels of stress. Marijuana mostly affects psychological well-being by its calming effects and sedative properties, elevates mood, and also helps in improving interpersonal relationships [1,2]. Studies potentially point to a complicated and causal relationship between marijuana consumption and depressive disorders, stating that depressive symptoms in the early stage enable marijuana usage, which then improves depressive symptoms [2]. The lack of research about this topic is unexpected given that those who consume marijuana commonly describe alleviation from depressive symptoms as the main motivation for using the drug and also because some studies suggest that depression predicts higher levels of marijuana usage in youth, and hence individuals with depressive disorders usually indulge in chronic marijuana consumption and abuse [26,27]. According to a cross-sectional study, from 2015 to 2016, 30 out of 100 individuals suffering from depressive disorders admitted to using marijuana in the last one month, and more than 25% of them reported daily marijuana consumption [7]. As illustrated in one of the cohort studies, individuals who consume marijuana for elevating mood and its euphoriant effects are undoubtedly linked with DSM-5 cannabis use disorder (CUD) [6].

Long-Term Effects of Marijuana

A research article clearly states that many studies indicate consuming marijuana can be helpful in elevating mood and reducing anxiety initially, but it is subsequently followed by a rise in depressive symptoms, which manifest as mental distress and frustration [28]. Moreover, a cross-sectional study indicated a link between recreational marijuana usage in the previous 30 days and depressive disorder. Researchers also found a more significant association between depressive disorders and regular marijuana consumption than between depressive disorders and occasional marijuana consumption [7]. A study conducted in 2013, after regulating for baseline determinants of MDD and marijuana use disorders, displayed conclusive proof of a reciprocal association between marijuana consumption and depressive disorders, proposing that a baseline CUD anticipated severe depression after 36 months and a baseline severe depressive disorder anticipated a later CUD [25]. Figure 6 shows the vicious cycle of marijuana consumption.

**Figure 4:**
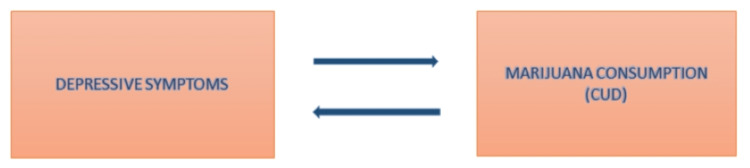
Vicious cycle of marijuana consumption CUD: cannabis use disorder Image credits: Prathma Anandbhai Dave, Priyansh Patel, and Ralph Kingsford Rohit

A systematic analysis done in 2014 suggested that when baseline depression was controlled for, marijuana consumption (particularly excessive use) was related to a higher likelihood of developing MDD in later stages [26]. Moreover, there are differences in the effect of marijuana on mental health depending on gender. A research article stated that in grown-ups, the relationship between marijuana dependency and the development of MDD was almost doubled for females as compared to males. Possible explanations for these disparities between men and women involve more neurological damage of substances in the female population due to petiteness, variations in duration for neurological development and influence of substances on the nervous system due to variations in hormones, and striking differences of societal abutment and expectations by gender [3]. In a randomized controlled trial and a recent study, Gage et al. concluded that if cannabis is used regularly, it may actually aggravate the symptoms of depression [1,24]. However, a prospective cohort study and a research article do not indicate the causal association between marijuana consumption and the development of depressive disorders [10,24]. As illustrated in a research article, patients with depressive symptoms who consume marijuana may be a specifically susceptible population for marijuana-related negative outcomes, such as worsening symptoms of depression and suicidal behavior. Discussions with patients about the extent of their marijuana consumption, techniques for reducing its use, and its impact on depression may be beneficial in medical facilities where depressive disorders are treated [7].

Limitations

There are few constraints in our research article that should be acknowledged. The availability of reliable evidence particularly through randomized controlled trials and meta-analyses is limited. As described in the methodology, only the articles that were free were included in this paper, and hence the papers that were not free were not assessed. Moreover, only the articles in the English language were included, and therefore information from the articles in languages other than English was not included in this paper. In addition, only the studies done on humans were included, and information from studies with animal trials was not included. All the studies that were included displayed diversity in the number of participants and quantification of determinants. Furthermore, the confounding factors associated with the trajectory of depression and marijuana consumption, such as alcohol and tobacco use, were not considered and might influence the results. Hence, more studies regulating these factors are needed to further strengthen the association between between marijuana consumption and depressive disorders.

## Conclusions

This research paper explored the influence of marijuana consumption on the risk of developing and worsening of depressive symptoms. It highlights the importance of understanding depression and the use of marijuana for temporary relief from depressive symptoms and also its long-term consequences on mental health. This article indicates a complicated and nuanced association between marijuana and depression and hence makes research on this topic indispensable. It is important for current practitioners and future researchers, as with the increasing legalization and easy availability of marijuana, there is an increasing trend of medical use of marijuana for the alleviation of depressive symptoms acutely. However, the long-term consequences of using marijuana medically should also be considered.

This research paper suggests that marijuana with the help of its psychoactive components, especially cannabidiol, has anxiolytic and euphoriant properties, temporarily lifting the mood. Meanwhile, chronic marijuana users have demonstrated an increased risk of developing or worsening depressive symptoms by causing alterations in the brain structure and disrupting the balance of neurotransmitters. Given the complex nature of association, it is crucial to investigate individual variations in susceptibility to marijuana's effects on depression, taking into consideration variables, such as heredity, co-occurring mood disorders, alcohol and tobacco use, dosage patterns, and other demographic factors. Moreover, future research should look at the possible medicinal usage of specific components of marijuana that might exert a more directed and advantageous influence on depressive symptoms sans the hazards associated with chronic consumption.
